# Knock-in mice harboring a Ca^2+^ desensitizing mutation in cardiac troponin C develop early onset dilated cardiomyopathy

**DOI:** 10.3389/fphys.2015.00242

**Published:** 2015-08-27

**Authors:** Bradley K. McConnell, Sonal Singh, Qiying Fan, Adriana Hernandez, Jesus P. Portillo, Peter J. Reiser, Svetlana B. Tikunova

**Affiliations:** ^1^Department of Pharmacological and Pharmaceutical Sciences, University of HoustonHouston, TX, USA; ^2^Division of Biosciences, College of Dentistry, The Ohio State UniversityColumbus, OH, USA

**Keywords:** troponin C, dilated cardiomyopathy, cardiac muscle, calcium, knock-in mouse

## Abstract

The physiological consequences of aberrant Ca^2+^ binding and exchange with cardiac myofilaments are not clearly understood. In order to examine the effect of decreasing Ca^2+^ sensitivity of cTnC on cardiac function, we generated knock-in mice carrying a D73N mutation (not known to be associated with heart disease in human patients) in cTnC. The D73N mutation was engineered into the regulatory N-domain of cTnC in order to reduce Ca^2+^ sensitivity of reconstituted thin filaments by increasing the rate of Ca^2+^ dissociation. In addition, the D73N mutation drastically blunted the extent of Ca^2+^ desensitization of reconstituted thin filaments induced by cTnI pseudo-phosphorylation. Compared to wild-type mice, heterozygous knock-in mice carrying the D73N mutation exhibited a substantially decreased Ca^2+^ sensitivity of force development in skinned ventricular trabeculae. Kaplan-Meier survival analysis revealed that median survival time for knock-in mice was 12 weeks. Echocardiographic analysis revealed that knock-in mice exhibited increased left ventricular dimensions with thinner walls. Echocardiographic analysis also revealed that measures of systolic function, such as ejection fraction (EF) and fractional shortening (FS), were dramatically reduced in knock-in mice. In addition, knock-in mice displayed electrophysiological abnormalities, namely prolonged QRS and QT intervals. Furthermore, ventricular myocytes isolated from knock-in mice did not respond to β-adrenergic stimulation. Thus, knock-in mice developed pathological features similar to those observed in human patients with dilated cardiomyopathy (DCM). In conclusion, our results suggest that decreasing Ca^2+^ sensitivity of the regulatory N-domain of cTnC is sufficient to trigger the development of DCM.

## Introduction

Dilated cardiomyopathy (DCM) is a progressive disease of the myocardium characterized by dilation and impaired systolic function of left or both ventricles (for review Wexler et al., 2009; Jefferies and Towbin, 2010). DCM frequently progresses to heart failure, and can result in sudden cardiac death. While DCM can be caused by a variety of factors, mutations in over 40 genes account for ~25–30% of cases (for review Towbin, 2014; McNally et al., 2015). Most of the genetic cases of DCM are inherited in an autosomal dominant pattern. An estimated 35–40% of genetic DCM cases are linked to mutations in genes encoding sarcomeric proteins, including all three subunits of the cardiac troponin (cTn) complex (for review Chang and Potter, 2005; McNally et al., 2015). Understanding how these mutations trigger pathogenesis of DCM is lacking, although DCM-linked mutations of sarcomeric proteins tend to desensitize myofilaments to Ca^2+^ (for review Kimura, 2010; Willott et al., 2010; Lu et al., 2013; Spudich, 2014).

The heterotrimeric cTn complex, consisting of cardiac troponin C (cTnC), cardiac troponin I (cTnI) and cardiac troponin T (cTnT), plays a key role in the regulation of muscle contractility (for review Farah and Reinach, 1995; Filatov et al., 1999; Parmacek and Solaro, 2004). cTnC, the Ca^2+^ sensing subunit of the cTn complex, consists of the N- and C-terminal globular domains linked by a central α-helix. Each globular domain contains a pair of EF-hand (29 residue helix-loop-helix) Ca^2+^ binding motifs, numbered I–IV. A canonical EF-hand motif consists of a 12 residue loop flanked by two α-helices (for review Nelson and Chazin, 1998). Coordination of Ca^2+^ involves six residues located in chelating positions 1(+X), 3 (+Y), 5 (+Z), 7 (−Y), 9 (−X), and 12 (−Z) of the loop. Due to inability of the first EF-hand to bind Ca^2+^, the second EF-hand controls Ca^2+^ binding and exchange with the regulatory N-domain of cTnC. The ability of cTnC to sense and respond to changes in intracellular Ca^2+^ is modulated by the other myofilament proteins. Mutations and post-translational modifications of myofilament proteins, linked to diseases of the myocardium, can result in an abnormal response of cTnC to Ca^2+^ (for review Davis and Tikunova, 2008).

CTnI, the inhibitory subunit of the cTn complex, transmits Ca^2+^ signal from cTnC to other components of the myofilaments (for review Farah and Reinach, 1995; Filatov et al., 1999; Parmacek and Solaro, 2004). Phosphorylation of cTnI by protein kinase A (PKA) on Ser^22/23^ residues, which occurs during β-adrenergic stimulation, desensitizes myofilaments to Ca^2+^ by accelerating the rate of Ca^2+^ dissociation from the regulatory N-domain of cTnC (for review Solaro et al., 2008; Solaro and Kobayashi, 2011; Solaro et al., 2013). Recent studies showed that a number of mutations in myofilament proteins, linked to cardiomyopathies, blunt or abolish the Ca^2+^ desensitizing effect of cTnI phosphorylation on Ser^22/23^ residues (for review Messer and Marston, 2014). Blunting the extent of Ca^2+^ desensitization induced by cTnI phosphorylation appears to be a common feature among DCM-linked mutations (for review Messer and Marston, 2014; Kalyva et al., 2014).

The overriding goal of our work is to gain a deeper insight into physiological consequences of altering Ca^2+^ binding and exchange with cardiac myofilaments. The objective of this work was to determine whether decreasing Ca^2+^ sensitivity of the regulatory N-domain of cTnC triggers the pathogenesis of DCM. To achieve this objective, we engineered a Ca^2+^ desensitizing mutation, not known to be associated with DCM in humans, into the regulatory N-domain of cTnC, and knocked this mutation into the mouse endogenous TNNC1 gene via gene targeting technology. Our results show that knock-in mice expressing Ca^2+^ desensitizing mutant of cTnC develop the phenotype of early onset DCM.

## Materials and methods

### Protein mutagenesis and purification

The cTnC^D73N^ and cTnI^S22D/S23D^ mutants were generated as previously described and verified by DNA sequencing (Tikunova et al., 2010). The cTn complex subunits were expressed, purified, and quantified as previously described (Albury et al., 2012; Swindle et al., 2014). Rabbit fast skeletal actin and bovine cTm were a generous gift from Dr. Darl Swartz (Delaware Valley College, Doylestown, PA).

### Fluorescent labeling of cTnC and cTnC^D73N^

The cTnC construct used to reconstitute the cTn complexes or thin filaments contained C35S, T53C, and C84S substitutions, to allow fluorescent labeling of cTnC at Cys^53^. CTnC and its mutant were labeled with the environmentally sensitive thiol-reactive probe IAANS at Cys^53^ as previously described (Davis et al., 2007).

### Reconstitution of the cTn complexes and thin filaments

The cTn complexes and thin filaments were prepared and reconstituted as previously described (Albury et al., 2012; Swindle et al., 2014).

### Determination of Ca^2+^ binding sensitivities and dissociation kinetics

Steady-state fluorescence measurements were carried out using a Perkin-Elmer LS55 fluorescence spectrometer at 15°C, as previously described (Albury et al., 2012; Swindle et al., 2014). Data were fit with a logistic sigmoid function mathematically equivalent to the Hill equation. Data represent a mean of at least three titrations ± S.E. All kinetic measurements were performed using an Applied Photophysics Ltd. (Leatherhead, UK) model SX.18MV stopped-flow instrument with a dead time of ~1.4 ms at 15°C, as previously described (Albury et al., 2012; Swindle et al., 2014). Data represent an average of at least three separate experiments ± S.E., each averaging at least five traces fit with a single exponential equation.

### Generation of knock-in mouse model

The Gene Targeting Mouse Service Core at the University of Cincinnati (Cincinnati, OH) generated the targeting vector and the knock-in mice. Mice were backcrossed into C57BL/6J background using Speed Congenic Service offered by the Jackson Laboratory (Bar Harbor, ME). The Institutional Animal Care and Use Committee (IACUC) approved all animal studies. Male mice, between 4 and 12 week old, were used in all experiments.

### Trabecula force vs. pCa measurements

Mice were euthanized by CO_2_ inhalation, followed by rapid cardiectomy, in accordance with an Ohio State University IACUC-approved protocol. The heart was placed in cold relaxing solution and swirled to remove blood from the chambers. All of the solutions were as previously described (Reiser et al., 2013). A single cut was made across the free wall of both ventricles and each heart was placed individually in a 30 ml plastic bottle containing glycerinating solution for storage at −20°C (1–4 weeks for both genotypes). Each day, on which force measurements were conducted, a heart was removed from glycerinating solution and was transferred to cold relaxing solution with 1% (v/v) Triton X-100. The heart was kept in this solution for 30–40 min on ice, with occasional gentle swirling. The heart was then transferred to a Sylgard-lined Petri dish containing relaxing solution and a single trabecula was isolated by dissection from either the right or left ventricle and mounted in the experimental chamber, as previously described (Reiser et al., 2013). Half of the trabeculae were from each ventricle for both genotypes. The trabecula was set to a length that was just beyond slack length and the trabecula was imaged using a digital camera. Resting sarcomere length was measured, with image analysis software, in a subset (three per each genotype) of the trabeculae in which a clear striation pattern could be observed. The length spanning a series of ~20 striations was measured. The sarcomere length in these trabeculae [2.18 ± 0.05 μm for WT mice vs. 2.2 ± 0.2 μm for heterozygous knock-in mice (termed D73N(+/−) mice)] did not differ significantly between genotypes. The width and depth of each trabecula were measured as previously described (Reiser et al., 2013). Cross-sectional area was calculated from the width and depth measurements, assuming an ellipsoidal cross-section. The trabecula was exposed to series of pCa (-log [Ca^2+^]) solutions, with complete relaxation (in pCa 9.0 solution) between each activation. Resting force was measured (in pCa 9.0 solution) and was subtracted from the total peak force during each activation to calculate peak active force. Every third activation was performed with pCa 4.0 solution and the force in that solution was used to normalize the force during each temporally neighboring submaximal activation. All force measurements were made at 15°C. There was a small decrement in force generating ability during the course of the measurements in each trabecula. This was quantitated by calculating the ratio of the force in the final activation with pCa 4.0 by the force generated in the initial activation with the same pCa. This ratio [0.88 ± 0.02 for WT mice vs. 0.94 ± 0.04 for D73N (+/−) mice] did not differ significantly between genotypes.

### Heart weight to body weight analysis

Mice were weighed and anesthetized with 2% isoflurane. Whole hearts were excised, rinsed with phosphate buffered saline to remove blood, blotted dry, and weighed.

### Histological analysis

Hearts were excised from anesthetized mice, and fixed in 10% buffered formalin. Paraffin-embedded longitudinal sections of whole mouse hearts were prepared and stained with hematoxylin and eosin (H&E) or Masson's trichrome by American HistoLabs, Inc (Gaithersburg, MD). In order to calculate myocardial fibrosis, the bright field images of longitudinal heart sections stained with Masson's trichrome were taken with Nikon Eclipse Ti. The fibrosis areas (blue) were measured by Image-Pro Plus software [Media Cybernetics, Inc. (Rockville, MD)]. The percentage of total fibrosis in each heart was calculated as the sum of blue stained areas divided by the whole ventricular area. Cross-sectional area of ventricular myocytes was measured from the images of H&E stained longitudinal heart sections using ImageJ software [NIH (Bethesda, MD)]. Data were obtained by measuring cross-sectional areas of at least 100 nucleated left ventricular myocytes per heart.

### Genotyping

Mouse genomic DNA was isolated from tail biopsies using Wizard SV Genomic DNA Purification Kit [Promega (Madison, WI)], according to manufacturer's instructions. Fragments of mouse genomic DNA were amplified by a PCR assay (termed P1, P2, P3 assay) using P1 (5′- AGGACTTGCTTGGCTTCTGAC), P2 (5′- AGGGTGTCTACCCGAAACTAC), P3 (5′-CTGCCATAGCCACTCGAGAAG) primer mix. The P1, P2, P3 assay was used to identify WT, heterozygous neo (+/−), heterozygous D73N (+/−), and homozygous D73N (+/+) mice. Product lengths were 384, 287, and 517 base pairs for WT, neo, and the D73N alleles, respectively.

### RT-PCR analysis

Total RNA was extracted from the left ventricular mouse tissues using an RNeasy Fibrous Tissue Mini Kit [QIAGEN (Venlo, Limburg)] according to the manufacturer's instructions. Fragments of cDNA were amplified from total RNA using Superscript III One Step RT PCR system [Invitrogen (Carlsbad, CA)], according to manufacturer's instruction. Ratio of the cTnC^D73N^ mutant to total cTnC in the D73N (+/−) mice was estimated by RT-PCR using TNNC1 sequence-specific primers to amplify a 349 bp region of DNA from total RNA isolated from the left ventricle of D73N (+/−) mice. The RT-PCR product was digested by the restriction enzyme KpnI. Densitometric analysis of RT-PCR gel image was performed using ImageJ software. The densities of the amplicon band before and after digestion with KpnI were used to calculate the fraction of cTnC^D73N^ in the D73N (+/−) mice. Sequences of primer sets used to amplify fragments of cDNA for cTnC, β-MHC (β-myosin heavy chain), α-MHC (α-myosin heavy chain), and GAPDH (glyceraldehyde-3-phosphate dehydrogenase) are available upon request.

### SDS-PAGE analysis

Left ventricular free wall samples were isolated by dissection and were prepared for analysis by SDS-PAGE. The protocols for sample preparation, gel preparation and running, gel staining, and densitometric scanning were as previously described (Reiser and Moravec, 2014). The relative amount of β-MHC [% of total MHC (α-MHC + β-MHC)] in each sample was determined from densitometric scans.

### Western blot analysis

Western blots were carried out as previously described (McConnell et al., 2009; Guillory et al., 2013). Briefly, left ventricles were dissected and flash frozen in liquid nitrogen. Proteins from left ventricular mouse tissue homogenates were separated by the SDS-PAGE gradient (4–12% Bis-Tris) gels, followed by immunoblotting. Primary antibodies against cTnI, cTnI^phospho^, GAPDH, and secondary antibody were purchased from Cell Signaling Technology (Beverly, MA). Primary antibody against cTnC was purchased from Abcam (Cambridge, England). Densitometric analysis of immunoblots was performed using ImageJ software.

### Echocardiography

Echocardiographic studies on mice anesthetized with 1% isoflurane were performed in the Mouse Phenotyping Core (Baylor University College of Medicine, Houston, TX) using VisualSonics Vevo 770 High-Resolution *In Vivo* Micro-Imaging System equipped with a 30 MHz microprobe (VisualSonics Inc.,Toronto, Ontario, Canada) as previously described (Guillory et al., 2013).

### Electrocardiography

Electrocardiographic (ECG) signals from mice anesthetized with 2% isoflurane were acquired and analyzed using Power Lab System with LabChart software (ADInstruments, Colorado Springs, CO). ECGs were recorded using subcutaneously inserted needle electrodes.

### Isolation of mouse ventricular myocytes

Adult mouse ventricular myocytes were isolated following a previously described protocol (Liao and Jain, 2007). Intracellular Ca^2+^ and contractility were measured at 37°C in isolated myocytes loaded with Fura 2-AM in the absence or presence of isoproterenol (ISO) (1 μmol/L) using an IonOptix system (Milton, MA), as previously described (Guillory et al., 2013). Myocytes were paced at 1 Hz with IonOptix MyoPacer Field stimulator (pulse duration 1 ms; 5 volts). Data were analyzed using IonWizard (IonOptix) software.

### Statistical analysis

Statistical significance was determined by an unpaired two-sample *t*-test using the statistical analysis software Minitab (State College, PA). The two means were considered to be significantly different when the *p*-value was < 0.05. All data are reported as mean value ± S.E. Kaplan-Meier survival curves (for review Rich et al., 2010) were analyzed by the log-rank test using GraphPad Prism software (La Jolla, CA).

## Results

### Effect of the D73N mutation on the Ca^2+^ sensitivity and the rate of Ca^2+^ dissociation from reconstituted thin filaments in the absence and presence of cTnI pseudo-phosphorylation

**Figure 1:**
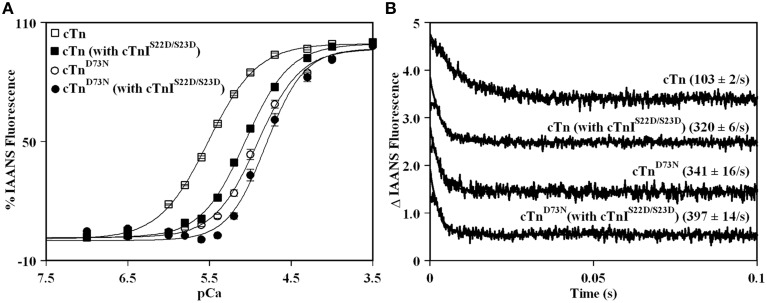
**Effect of the D73N mutation on the Ca^2+^ sensitivity and the rate of Ca^2+^ dissociation from reconstituted thin filaments in the absence and presence of cTnI pseudo-phosphorylation. (A)** Increases in IAANS fluorescence, which occur as Ca^2+^ binds to the regulatory N-domain of the cTn complex, containing cTnI (□) or cTnI^S22D/S23D^ (■), reconstituted into thin filaments; and to the regulatory N-domain of the cTn^D73N^ complex, containing cTnI (◦) or cTnI^S22D/S23D^ (•), reconstituted into thin filaments. Data represent the mean ± S.E. of at least three titrations fit with logistic sigmoid function. IAANS fluorescence was excited at 330 nm and monitored at 450 nm. **(B)** Time course of decreases in IAANS fluorescence as Ca^2+^ was removed by excess EGTA from the regulatory N-domain of the cTn complex, containing cTnI or cTnI^S22D/S23D^, reconstituted into thin filaments; and from the regulatory N-domain of the cTn^D73N^ complex, containing cTnI or cTnI^S22D/S23D^, reconstituted into thin filaments. The data traces have been normalized and staggered for clarity. Each trace is an average of at least five traces fit with single exponential equation. The IAANS fluorescence was excited at 330 nm and monitored through a 510 nm bandpass filter.

Previously, we demonstrated that Ca^2+^ binding and exchange with an EF-hand within a Ca^2+^ binding protein can be altered by mutations of the chelating loop residues (Black et al., 2000; Liu et al., 2012a). Based on the earlier studies (Black et al., 2000; Liu et al., 2012a), the D73N mutation (substitution of an acidic Asp in the -X position of the second Ca^2+^ binding loop with neutral Asn) was engineered into cTnC in order to desensitize the regulatory N-domain of cTnC to Ca^2+^. The Ca^2+^ induced increases in IAANS fluorescence, occurring when Ca^2+^ binds to thin filaments reconstituted with the cTn or cTn^D73N^ complex, are shown in Figure 1A and are summarized in Table 1. Thin filaments reconstituted with the cTn complex exhibited a half-maximal Ca^2+^ dependent increase in IAANS fluorescence with a pCa_50_ of 5.45 ± 0.06. The D73N mutation led to substantial Ca^2+^ desensitization of reconstituted thin filaments (ΔpCa_50_ = −0.55), without affecting Ca^2+^ binding cooperativity (as indicated by the Hill coefficient n_H_).

**Table 1:** **Effect of the D73N mutation on the Ca^2+^ sensitivity and the rate of Ca^2+^ dissociation from reconstituted thin filaments in the absence and presence of cTnI pseudo-phosphorylation**.

**Parameter**	**cTn**	**cTn (with cTnI^S22D/S23D^)**	**cTn^D73N^**	**cTn^D73N^ (with cTnI^S22D/S23D^)**
pCa_50_	5.45±0.06	5.06±0.03^†^	4.90±0.05^*^	4.78±0.06^‡^^≠^
n_H_	1.45±0.5	1.6±0.1	1.68±0.08	2.2±0.8^‡^^#^^≠^
Ca^2+^ k_off_ (s^−1^)	103±2	320±6^†^	341±16^*^	397±14^‡^^#^^≠^

*All values are expressed as mean ± S.E. for at least three experiments per group*.

* cTn^D73N^ values significantly different from their respective cTn values;

† cTn (with cTnI^S22D/S23D^) values significantly different from their respective cTn values;

‡ cTn^D73N^ (with cTnI^S22D/S23D^) values significantly different from their respective cTn (with cTnI^S22D/S23D^) values;

# cTn^D73N^ (with cTnI^S22D/S23D^) values significantly different from their respective cTn^D73N^ values;

≠ *cTn^D73N^ (with cTnI^S22D/S23D^) values significantly different from their respective cTn values. The p-value < 0.05 was considered statistically significant*.

Figure 1A also shows the Ca^2+^ induced increases in IAANS fluorescence that occur when Ca^2+^ binds to thin filaments reconstituted with the cTn or cTn^D73N^ complex, containing the phosphomimetic of cTnI (cTnI^S22D/S23D^). Thin filaments reconstituted with the cTn complex, containing cTnI^S22D/S23D^, exhibited a half-maximal Ca^2+^ dependent increase in IAANS fluorescence with a pCa_50_ of 5.06 ± 0.03. In the presence of cTnI pseudo-phosphorylation, the D73N mutation still led to Ca^2+^ desensitization of reconstituted thin filaments (ΔpCa_50_ = −0.28).

Our results indicate that replacement of cTnI by the phosphomimetic of cTnI resulted in a substantial Ca^2+^ desensitization (ΔpCa_50_ = −0.39) of thin filaments reconstituted with the cTn complex. However, replacement of cTnI by the phosphomimetic of cTnI resulted in only minor Ca^2+^ desensitization (ΔpCa_50_ = −0.12) of thin filaments reconstituted with the cTn^D73N^ complex. Thus, our results indicate that the D73N mutation drastically blunted the extent of Ca^2+^ desensitization (of thin filaments reconstituted with the cTn^D73N^ complex) induced by cTnI pseudo-phosphorylation.

Fluorescence stopped-flow measurements, utilizing IAANS fluorescence, were conducted to determine the effect of the D73N mutation in cTnC on the rate of Ca^2+^ dissociation from the regulatory N-domain of the cTn complex reconstituted into thin filaments in the absence and presence of cTnI pseudo-phosphorylation. The results are summarized in Table 1. Figure 1B shows that Ca^2+^ was removed (by excess EGTA) from thin filaments reconstituted with the cTn or cTn^D73N^ complex at a rate of 103 ± 2, or 341 ± 16 s^−1^. Thus, the D73N mutation led to a substantial ~3.3-fold increase in the rate of Ca^2+^ dissociation from thin filaments reconstituted with the cTn complex. Figure 1B also shows that Ca^2+^ was removed (by excess EGTA) from thin filaments reconstituted with the cTn or cTn^D73N^ complex, containing phosphomimetic of cTnI, at a rate of 320 ± 6, or 397 ± 14 s^−1^. Thus, in the presence of cTnI pseudo-phosphorylation, the D73N mutation led to only ~1.2-fold faster rate of Ca^2+^ dissociation from reconstituted thin filaments.

Our results indicate that replacement of cTnI by the phosphomimetic of cTnI led to ~3.1-fold increase in the rate of Ca^2+^ dissociation from thin filaments reconstituted with the cTn complex. On the other hand, replacement of cTnI by the phosphomimetic of cTnI led to only ~1.2-fold increase in the rate of Ca^2+^ dissociation from thin filaments reconstituted with the cTn^D73N^ complex. Thus, our results indicate that the D73N mutation blunted the effect of cTnI pseudo-phosphorylation on the rate of Ca^2+^ dissociation from thin filaments reconstituted with the cTn^D73N^ complex.

### Generation of knock-in mice

**Figure 2:**
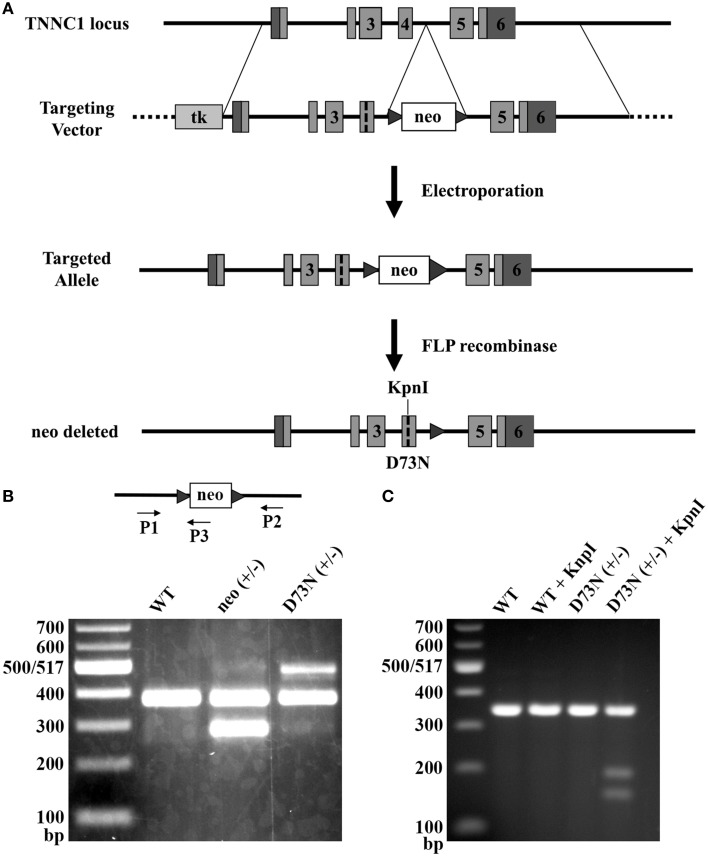
**Generation of knock-in mice. (A)** The gene targeting strategy to introduce the D73N mutation in cTnC into mouse myocardium. Targeting vector harbored thymidine kinase (tk) gene, neo cassette flanked by FRT sites in intron 4, the D73N mutation and two silent substitutions in order to create a restriction site for the enzyme KpnI in exon 4. Targeting vector was introduced into ES cells by electroporation. Neo cassette was deleted by crossing neo (+/−) mice with mice expressing FLP recombinase to create the D73N (+/−) mice. **(B)** PCR mediated genotyping of WT, neo (+/−), and the D73N (+/−) mice. Product lengths are 384, 287, and 517 base pairs for WT, neo, and D73N alleles, respectively. **(C)** Undigested and digested RT-PCR products for WT and the D73N (+/−) mice. KpnI restriction site was present in the D73N (+/−) mice but not in WT mice. Total RNA was isolated from the left ventricle of four hearts per genotype, with each KpnI digestion conducted in triplicate.

Due to its Ca^2+^ desensitizing properties, the D73N mutation in cTnC was selected for the introduction into the TNNC1 gene. Figure 2A illustrates the gene targeting strategy used to change the GAC codon to an AAC codon in exon 4 of the TNNC1 gene, which corresponds to the D73N mutation in cTnC. In addition to the D73N mutation, the targeting vector harbored two silent substitutions in exon 4 in order to generate a restriction site for the enzyme KpnI. The targeting vector also harbored a floxed neomycin (neo) resistance cassette in intron 4. Linearized targeting vector was injected into 129SvEv ES cells by electroporation. Two ES cell clones, which integrated the construct by homologous recombination, were identified by PCR and confirmed by Southern blotting (data not shown). Properly targeted ES cell clones were injected into C57BL/6 recipient blastocysts to produce chimeras. Male chimeras were crossed with C57BL/6J females to obtain heterozygous mice with one allele carrying the D73N mutation and the neo cassette, termed neo (+/−) mice. The neo (+/−) mice were crossed with FLP deleter mice, in order to remove the neo cassette, resulting in heterozygous mice (with the FLP transgene) carrying the D73N mutation in cTnC. Subsequent round of breeding with the C57BL/6J mice removed the FLP transgene, generating heterozygous mice carrying the D73N mutation in cTnC, termed D73N (+/−) mice. Removal of the neo cassette was confirmed by PCR (Figure 2B).

Genotyping of the D73N (+/−) mice and their WT littermates at 3 weeks revealed an expected Mendelian frequency. Figure 2C shows that restriction site for KpnI was present in the amplicon from the D73N (+/−) mice but not from WT mice. Because RNA was isolated from heterozygous D73N (+/−) mice, the band in the D73N (+/−) mice + KpnI, which lines up with the other groups, corresponds to the WT allele (since the WT allele doesn't have a restriction site for KpnI). The amount of cTnC^D73N^ transcript in the left ventricle of the D73N (+/−) mice was estimated at 31 ± 2% of total cTnC transcript. On the other hand, homozygous mice were born at ~ half of expected Mendelian ratio. None of the homozygous D73N (+/+) mice survived longer than 1 day, suggesting both embryonic and neonatal lethality.

### Effect of the D73N mutation on the Ca^2+^ sensitivity of force development in skinned ventricular trabeculae

**Figure 3:**
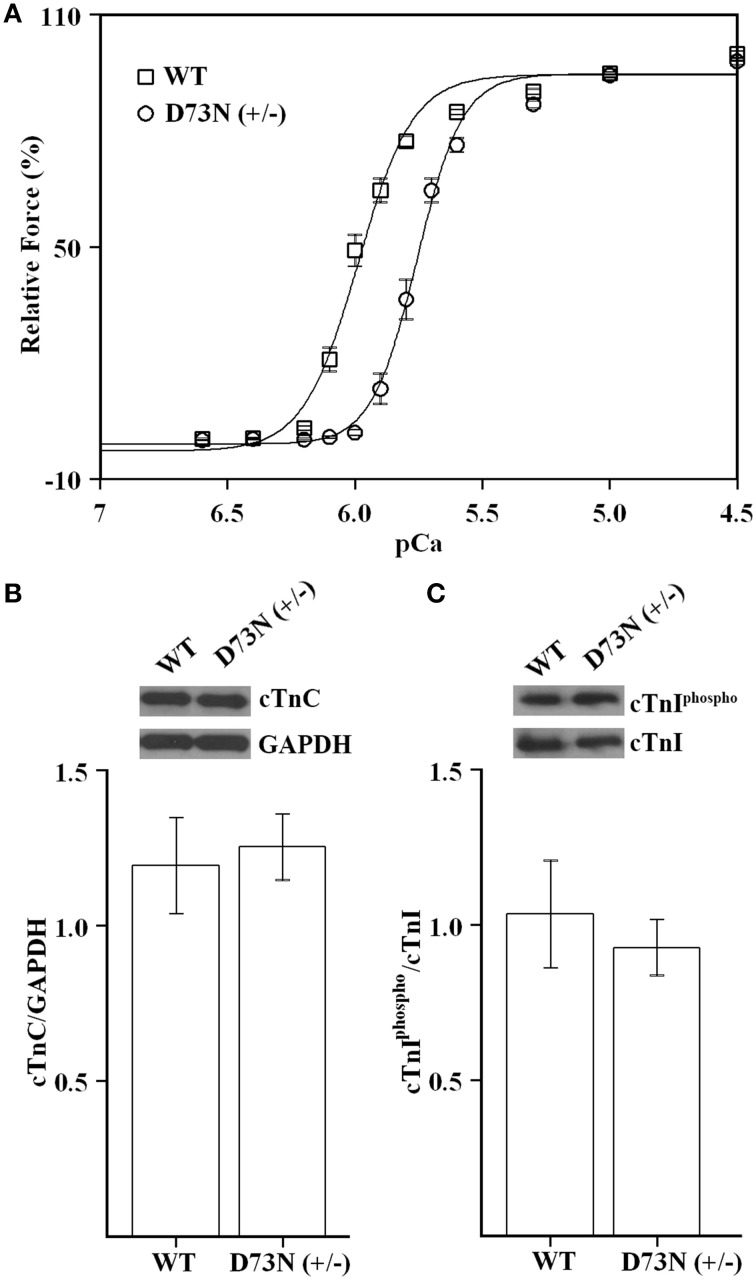
**Effect of the D73N mutation on the Ca^2+^ sensitivity of force development in skinned ventricular trabeculae. (A)** Ca^2+^ dependence of force development for skinned ventricular trabeculae isolated from 8 ± 0.6 week old WT (□) or D73N (+/−) (◦) mice. Data represent the mean ± S.E. for 10 trabeculae per genotype (1–2 trabeculae dissected per mouse heart). Data sets were individually normalized for each genotype and fit with logistic sigmoid. **(B)** Western blot analysis of total cTnC expression in the left ventricular tissue homogenates isolated from 8 ± 0.4 week old mice. The top panel shows a representative image of a Western blot probed with an antibody to cTnC or to GAPDH. The bar graphs show the ratio of total cTnC to GAPDH. Data represent the mean ± S.E. for four hearts per genotype. **(C)** Western blot analysis of cTnI phosphorylation in the left ventricular tissue homogenates isolated from 8 ± 0.4 week old mice. The top panel shows a representative image of a Western blot probed with an antibody to phosphorylated cTnI or to total cTnI. The bar graphs show the ratio of phosphorylated cTnI to total cTnI. Data represent the mean ± S.E. for four hearts per genotype.

Since the D73N mutation desensitized reconstituted thin filaments to Ca^2+^, we expected that ventricular trabeculae isolated from the D73N (+/−) mice would display reduced Ca^2+^ sensitivity of force development. Figure 3A shows that skinned ventricular trabeculae isolated from WT mice developed force with a pCa_50_ = 5.99 ± 0.01, while skinned ventricular trabeculae isolated from the D73N (+/−) mice developed force with a pCa_50_ = 5.76 ± 0.02, respectively. Thus, our results indicate that the D73N mutation led to a statistically significant decrease in the Ca^2+^ sensitivity of force development (ΔpCa_50_ = −0.23). The Hill coefficient values were not significantly different between WT and the D73N (+/−) mice [n_H_ = 4.6 ± 0.3 for WT mice vs. n_H_ = 5.3 ± 0.3 for the D73N (+/−) mice]. The D73N mutation did not significantly affect the maximum isometric force per cross-sectional area generated by the skinned cardiac trabeculae [*F*_max_ = 16 ± 2 kN/m^2^ for WT mice vs. *F*_max_ = 19 ± 3 kN/m^2^ for D73N (+/−) mice]. Western blot analysis confirmed that expression levels of total cTnC were not altered by the D73N mutation (Figure 3B). We also determined that the D73N mutation did not significantly affect the level of cTnI phosphorylation at Ser^22/23^ (Figure 3C).

### Effect of the D73N mutation on survival, heart weight to body weight ratios, overall cardiac morphology, and expression of a molecular marker of cardiac hypertrophy

**Figure 4:**
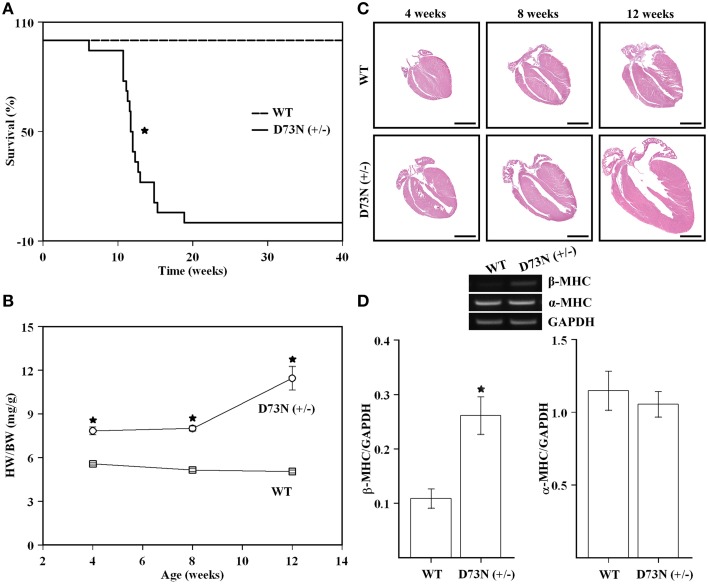
**Effect of the D73N mutation on survival, heart weight to body weight ratios, overall cardiac morphology, and expression of a molecular marker of cardiac hypertrophy. (A)** Kaplan-Meier survival curves for WT (*n* = 31) and D73N (+/−) (*n* = 18) mice. The log-rank test indicated statistically significant difference between the two curves (^*^p < 0.0001). **(B)** Comparison of heart weight to body weight ratios between 4 ± 0.1, 8 ± 0.4, and 12 ± 0.4 week old WT and D73N (+/−) mice. Data represent mean ± S.E. for at least seven mice per group. **(C)** Representative images of longitudinal heart sections of 4 ± 0.1, 8 ± 0.4, and 12 ± 0.4 week old WT and D73N (+/−) mice, stained with H&E. Scale bar = 2 mm. **(D)** RT-PCR analysis of total RNA isolated from the hearts of WT and D73N (+/−) mice. Total RNA was isolated from the left ventricular myocardium of 8 ± 0.4 week old mice and subjected to RT-PCR. The top panel shows representative images of amplification for β-MHC, α-MHC, or GAPDH mRNAs, respectively. The bar graphs show the ratio of β-MHC or α-MHC to GAPDH. Data represent the mean ± S.E. for four hearts per genotype. ^*^D73N (+/−) values significantly different from their respective WT values (*p* < 0.05).

The D73N (+/−) mice were viable, fertile, and displayed no obvious differences in appearance or activity levels compared to WT mice. However, the D73N (+/−) mice began dying at 6 weeks of age and were all dead by 19 weeks of age. Approximately 83% of mice died suddenly, the rest began exhibiting clear signs of distress and had to be euthanized. Kaplan-Meier survival analysis (Figure 4A) showed that median survival age of the D73N (+/−) mice was ~12 weeks. Between 4 and 12 weeks, no significant differences in body weight were observed between WT and the D73N (+/−) mice (data not shown). However, heart weights were significantly increased in the D73N (+/−) mice (data not shown), reflected by increases in heart weight to body weight ratios between 4 and 12 weeks of age (Figure 4B).

Figure 4C shows representative images of longitudinal sections of whole mouse hearts stained with H&E at 4, 8 or 12 weeks of age. Analysis of the images revealed a gradual increase in heart size and dilation of the left ventricular chamber in the D73N (+/−) mice. At 12 weeks, ~25% of hearts in the D73N (+/−) mice displayed left atrial thrombosis (not shown).

RT-PCR analysis was used to evaluate the effect of the D73N mutation in cTnC on expression of β-MHC and α-MHC in the left ventricle of 8 week old knock-in mice. Our results show that transcript levels of β-MHC, a marker of heart failure, were increased ~2.4-fold in the left ventricle of 8 week old mice, while transcript levels of α-MHC were not affected (Figure 4D). Results of RT-PCR were confirmed by the SDS-PAGE analysis, showing a statistically significant increase in β-MHC expression in the left ventricle of 8 week old D73N (+/−) mice at the protein level [β-MHC = 0.1 ± 0.1% of total MHC in WT mice vs. β-MHC = 3 ± 1% of total MHC in D73N (+/−) mice] (gel not shown).

### Effect of the D73N mutation on cardiac histology

**Figure 5:**
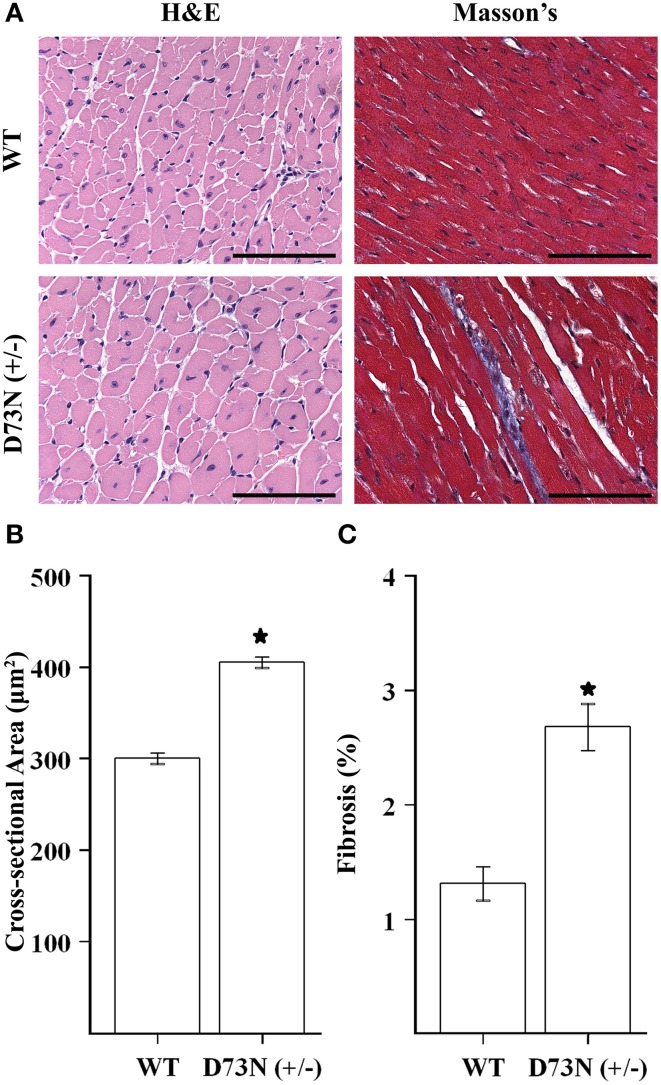
**Effect of the D73N mutation on cardiac histology. (A)** Representative microscopic images of left ventricular sections of 12 ± 0.4 week old WT and D73N (+/−) mice, stained with H&E and Masson's trichrome. Scale bar = 100 μm **(B)** Bar graphs show values for cross-sectional area of myocytes from the left ventricular free wall of 12 ± 0.4 week old WT and D73N (+/−) mice. Data represent the mean ± S.E. for at least 100 nucleated cells per heart from five hearts per genotype. **(C)** Bar graphs show the percentage of fibrotic area in the ventricles of 12 ± 0.4 week old WT and D73N (+/−) mice. Data represent the mean ± S.E. for five hearts per genotype. ^*^D73N (+/−) values significantly different from their respective WT values (*p* < 0.05).

We wanted to evaluate the effect of the D73N mutation in cTnC on cardiac histology. Figure 5A shows representative images of ventricular sections from 12 week old mice, stained with H&E or Masson's trichrome. Histological examination of H&E stained ventricular sections revealed that the D73N mutation led to ~35% increase in myocyte cross-sectional area (Figure 5B). In addition, histological analysis of Masson's trichrome stained ventricular sections revealed that the D73N mutation led to a small but statistically significant increase in myocardial fibrosis (Figure 5C).

### Effect of the D73N mutation on echocardiographic parameters

**Figure 6:**
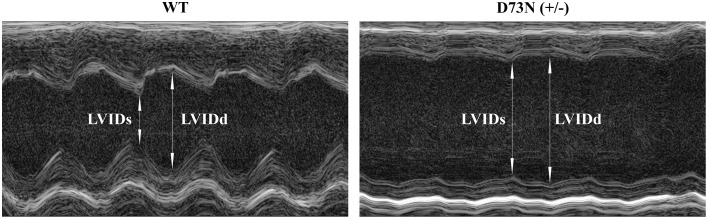
**Effect of the D73N mutation on echocardiographic parameters**. Representative transthoracic echocardiographic M-mode images of the left ventricle were obtained from 8 ± 0.4 week old WT and D73N (+/−) mice. Arrows indicate left ventricular internal dimensions in diastole (LVIDd) and systole (LVIDs). Values for LVIDd and LVIDs are listed in Table 2.

The effect of the D73N mutation in cTnC on cardiac dimensions and contractility of 8 week old mice was evaluated using echocardiography. Results are shown in Figure 6 and summarized in Table 2. Echocardiography demonstrated that the D73N mutation in cTnC did not significantly affect heart rate of knock-in mice. However, left ventricular posterior wall (LVPW) thickness in the D73N (+/−) mice was significantly decreased in systole, while left ventricular anterior wall (LVAW) thickness in the D73N (+/−) mice was significantly decreased in systole and diastole. In addition, left ventricular internal dimensions (LVID) of the D73N (+/−) mice were significantly increased in both systole and diastole. Furthermore, ejection fraction (EF) and fractional shortening (FS), measures of cardiac systolic function, were significantly reduced in the D73N (+/−) mice. Knock-in mice were able to maintain stroke volume (SV) due to increased LV dimensions. These results indicate that the Ca^2+^ desensitizing mutation in cTnC resulted in hallmark features of DCM in knock-in mice, namely left ventricular dilation, thinned left ventricular walls, and impaired systolic function.

**Table 2:** **Effect of the D73N mutation on echocardiographic parameters**.

**Parameter**	**WT**	**D73N /(+/−)**
BW (g)	21.2±0.5	21.1±0.4
HR (bpm)	477±16	449±26
LVIDd (mm)	3.7±0.2	5.2±0.1^*^
LVIDs (mm)	2.3±0.1	4.5±0.2^*^
LVAWd (mm)	0.99±0.03	0.85±0.03^*^
LVAWs (mm)	1.52±0.06	1.11±0.04^*^
LVPWd (mm)	0.82±0.06	0.67±0.03
LVPWs (mm)	1.27±0.09	0.79±0.04^*^
EF (%)	69±2	28±3^*^
FS (%)	38±2	13±2^*^
SV (μL)	41±4	34±2

*The WT and the D73N (+/−) mice were 8 ± 0.4 weeks old. All values are expressed as mean ± S.E. (n = 7–8 mice per genotype). BW, body weight; HR, heart rate; LVID, left ventricular internal dimensions; LVAW, left ventricular anterior wall; LVPW, left ventricular posterior wall; EF, ejection fraction; FS, fractional shortening; SV, stroke volume; d, diastole; s, systole*.

* *D73N (+/−) values significantly different from their respective WT values (p < 0.05)*.

### Effect of the D73N mutation on electrocardiographic parameters

**Figure 7:**
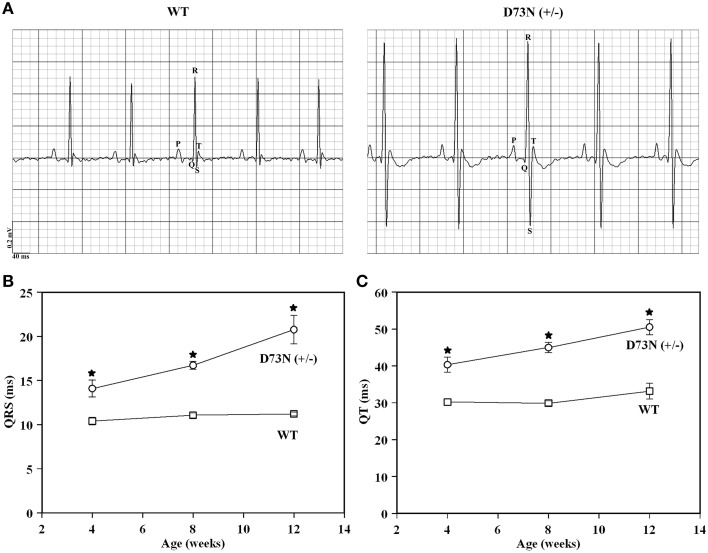
**Effect of the D73N mutation on electrocardiographic parameters. (A)** Representative ECG tracings from 8 ± 0.4 week old WT and D73N (+/−) mice. **(B)** Comparison of QRS intervals between 4 ± 0.1, 8 ± 0.4, and 12 ± 0.4 week old WT and D73N (+/−) mice. Data represent mean ± S.E. for at least 11 mice per group. **(C)** Comparison of QT intervals between 4 ± 0.1, 8 ± 0.4, and 12 ± 0.4 week old WT and D73N (+/−) mice. Data represent mean ± S.E. for at least 11 mice per group. ^*^D73N (+/−) values significantly different from their respective WT values (*p* < 0.05).

Electrocardiography (ECG) was utilized to assess the effect of the D73N mutation on the electrical activity of the heart. Figure 7A shows representative ECG tracings from 8 week old WT and the D73N (+/−) mice. At 8 weeks, the D73N mutation did not significantly affect heart rate [HR = 435 ± 7 bpm WT mice vs. HR = 449 ± 11 bpm in D73N (+/−) mice]. However, the D73N mutation led to a statistically significant increase in the QRS complex amplitude between 4 and 12 weeks of age (not shown), indicative of cardiac hypertrophy in the D73N (+/−) mice. In addition, the D73N mutation led to prolongation in QRS and QT intervals at 4 weeks of age, with further prolongation in QRS and QT intervals at 8 and 12 weeks of age (Figures 7B,C). Thus, our results indicate that the D73N mutation led to electrocardiographic abnormalities in knock-in mice.

### Effect of the D73N mutation on Ca^2+^ transients and contractility of ventricular myocytes

**Figure 8:**
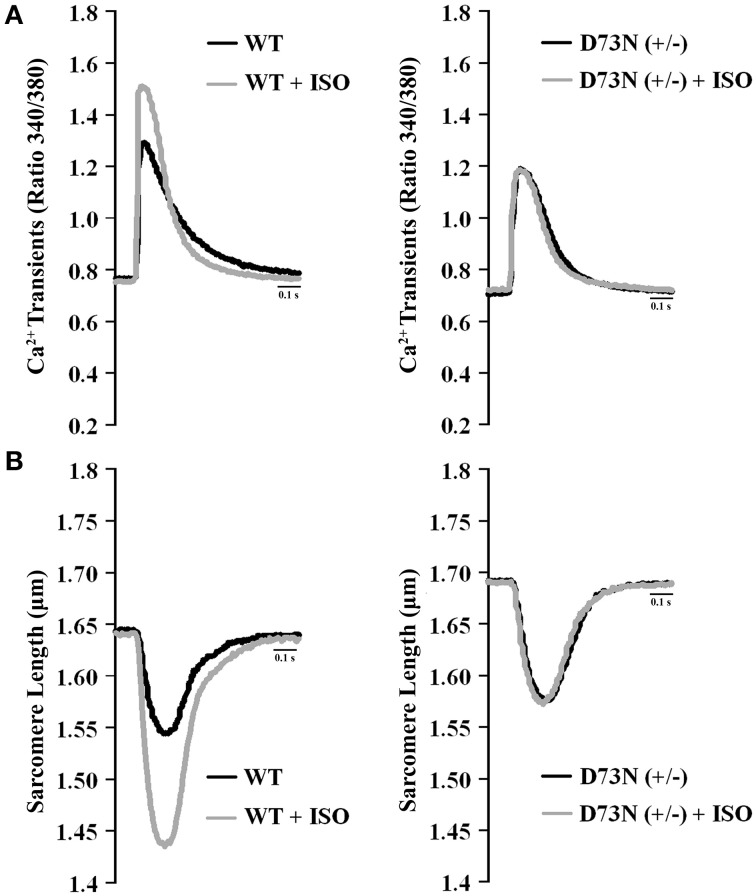
**Effect of the D73N mutation on Ca^2+^ transients and contractility of ventricular myocytes. (A)** Representative Ca^2+^ transients in ventricular myocytes paced at 1 Hz, isolated from 8 ± 0.7 week old WT and D73N (+/−) mice, before and after ISO perfusion. **(B)** Representative sarcomere length changes in ventricular myocytes paced at 1 Hz, isolated from 8 ± 0.7 week old WT and D73N (+/−) mice, before and after ISO perfusion.

We investigated whether the D73N mutation in cTnC resulted in altered Ca^2+^ transients and myocyte contractility. Figure 8 shows the effect of the D73N mutation on the Ca^2+^ transients (Figure 8A) and sarcomere shortening (Figure 8B) of Fura-2 loaded isolated ventricular myocytes. Results are summarized in Table 3. In the absence of ISO stimulation, resting sarcomere length of myocytes isolated from the D73N (+/−) mice was significantly longer than that in WT mice, consistent with reduced myofilament Ca^2+^ sensitivity in the hearts of the D73N (+/−) mice. In the absence of ISO stimulation, baseline Ca^2+^, the peak height of intracellular Ca^2+^ transient, departure velocity (maximal rate of change during Ca^2+^ release or shortening phase of the transient) and return velocity (maximal rate of change during the Ca^2+^ reuptake or re-lengthening phase of the transient) of Ca^2+^ transient/sarcomere shortening, and fractional sarcomere shortening were not significantly affected by the D73N mutation. An increase in resting sarcomere length could lead to an increase in fractional sarcomere shortening (Mullins and Bondarenko, 2013), compensating for lower myofilament Ca^2+^ sensitivity and resulting in normal sarcomere shortening in myocytes isolated from the D73N (+/−) mice. Similarly, fractional sarcomere shortening was not reduced in myocytes isolated from heterozygous knock-in mice harboring the DCM-linked ΔK210 mutation in cTnT (heterozygous ΔK210cTnT mice), despite reduced Ca^2+^ sensitivity of force development observed in these mice (Du et al., 2007).

**Table 3:** **Effect of the D73N mutation on Ca^2+^ transients and sarcomere length shortening measurements**.

**Parameter**	**WT**	**WT /+ ISO**	**D73N /(+/−)**	**D73N /(+/−)+ISO**
**Ca^2+^ TRANSIENT MEASUREMENTS**				
Baseline (R)	0.76±0.03	0.75±0.02	0.71±0.02	0.72±0.01
Peak height (R)	0.51±0.04	0.71±0.04^†^	0.48±0.03	0.52±0.03^‡^
Time to 50% peak (s)	0.016±0.001	0.0123±0.0004^†^	0.020±0.002^*^	0.018±0.001^‡^
Time to 75% peak (s)	0.021±0.001	0.0163±0.0005^†^	0.026±0.002^*^	0.023±0.001^‡^
Time to 50% baseline(s)	0.175±0.008	0.142±0.006^†^	0.177±0.008	0.164±0.006^‡^
Time to 75% baseline (s)	0.29±0.01	0.20±0.01^†^	0.24±0.01^*^	0.22±0.01^≠^
Departure velocity (R/s)	31±3	51±4^†^	28±3	27±2^‡^
Return velocity (R/s)	−2.7±0.3	−5.6±0.4^†^	−3.4±0.3	−4.0±0.4^‡^^≠^
**SARCOMERE LENGTH SHORTENING MEASUREMENTS**				
Resting SL length (μm)	1.64±0.02	1.64±0.02	1.70±0.01^*^	1.69±0.02^≠^
Peak height (μm)	0.100±0.009	0.20±0.01^†^	0.117±0.009	0.12±0.01^‡^
Fractional shortening (%)	6.1±0.5	12.3±0.8^†^	6.9±0.5	7.4±0.7^‡^
Time to 50% peak (s)	0.046±0.004	0.038±0.003	0.065±0.003^*^	0.059±0.003^‡^^≠^
Time to 75% peak (s)	0.071±0.006	0.055±0.005	0.088±0.004^*^	0.082±0.004^‡^
Time to 50% baseline (s)	0.27±0.03	0.22±0.02	0.29±0.01	0.27±0.01
Time to 75% baseline (s)	0.31±0.03	0.25±0.03	0.33±0.02	0.33±0.02^‡^
Departure velocity (μm/s)	−2.2±0.3	−4.8±0.5^†^	−1.8±0.2	−2.0±0.2^‡^
Return velocity (μm/s)	1.1±0.2	3.0±0.5^†^	1.0±0.1	1.2±0.3^‡^

*Ventricular myocytes were isolated from 8 ± 0.7 week old mice. Data values are expressed as mean ± S.E. (n = 14–19 cells isolated from three hearts per genotype). SL, sarcomere length; R, ratio 340/380. Time to 50% peak, time to 75% peak, time to 50% baseline and time to 75% baseline were measured from time 0*.

* D73N (+/−) values significantly different from their respective WT values;

† WT + ISO values significantly different from their respective WT values;

‡ D73N (+/−) + ISO values significantly different from their respective WT + ISO values;

≠ *D73N (+/−) + ISO values significantly different from their respective WT values. The p-value < 0.05 was considered statistically significant*.

Myocytes isolated from WT mice displayed increases in peak height of intracellular Ca^2+^ transient and fractional sarcomere shortening in response to ISO stimulation. In addition, myocytes isolated from WT mice exhibited faster departure and return velocities in response to ISO stimulation. However, myocytes isolated from the D73N (+/−) mice did not respond to ISO stimulation. In fact, peak height of intracellular Ca^2+^ transient and fractional sarcomere shortening were significantly reduced in myocytes isolated from the D73N (+/−) mice in the presence of ISO stimulation compared to that in WT mice. In addition, departure and return velocities of Ca^2+^ transient/sarcomere shortening were significantly slower in myocytes isolated from the D73N (+/−) mice in the presence of ISO stimulation compared to that in WT mice. Thus, our results indicate that the D73N mutation abolished the response to β-adrenergic stimulation at the cellular level.

## Discussion

The objective of this study was to determine whether desensitizing the regulatory N-domain of cTnC to Ca^2+^, by accelerating the rate of Ca^2+^ dissociation, affects the physiological function of the heart. At sub-saturating levels of Ca^2+^, the rate of cardiac muscle contraction is modulated by the Ca^2+^ binding properties of cTnC (Norman et al., 2007). Since the heart primarily functions at sub-saturating levels of Ca^2+^ (for review Davis and Tikunova, 2008), lower Ca^2+^ sensitivity of the regulatory N-domain of cTnC is expected to result in a decreased number of force-generating cross-bridges recruited during contraction, leading to systolic dysfunction. Dilation is expected to occur as a compensatory reaction of the heart to the weakened cardiac muscle.

Earlier studies showed the importance of acidic residues in chelating positions of an EF-hand in controlling Ca^2+^ binding and exchange with the protein (Procyshyn and Reid, 1994a,b; Wu and Reid, 1997; Black et al., 2000). In order to decrease the Ca^2+^ sensitivity of the regulatory N-domain of cTnC, an acidic Asp residue in the (-X) position of the second Ca^2+^ binding loop was substituted with neutral Asn residue, generating cTnC^D73N^. The D73N mutation decreased the Ca^2+^ sensitivity of the regulatory N-domain of cTnC reconstituted into the thin filament by increasing the rate of Ca^2+^ dissociation (Figure 1). The D73N mutation was then knocked into the endogenous TNNC1 gene via gene targeting technology (Figure 2), generating the D73N (+/−) mouse model. The advantage of a knock-in approach is that an endogenous promoter and regulatory elements are utilized to express the mutant protein, ensuring expression in the proper tissues (for review Snider and Conway, 2011).

Our results show that trabeculae isolated from the ventricles of the D73N (+/−) mice displayed a substantial decrease in the Ca^2+^ sensitivity of force development compared to WT mice, without affecting cooperativity or maximum force (Figure 3). The D73N (+/−) mice were viable and fertile, with no obvious differences in appearance, body weights or activity levels under normal mouse husbandry conditions. However, the D73N (+/−) mice developed hallmark features of early onset DCM, such as: (1) premature death (Figure 4), (2) increased heart weight to body weight ratios (Figure 4), (3) small but significant increase in myocardial fibrosis (Figure 5), (4) increased expression of β-MHC (Figure 4), (4) increased LVID with thinned left ventricular walls (Figure 6), (5) decreased EF and FS (Figure 6), and (6) electrophysiological abnormalities (Figure 7).

Diagnostic criteria for DCM in human patients include EF of less than 45% and/or FS of less than 25% (for review Taylor et al., 2006). Echocardiographic assessment of D73N (+/−) mice revealed an average EF of ~28% and FS of ~13% (Table 2), consistent with the diagnosis of DCM. Thus, our results indicate that the D73N mutation resulted in a substantial impairment of left ventricular systolic function.

The RT-PCR results revealed a marked increase in β-MHC transcript level in the left ventricle of the D73N (+/−) mice compared to that in WT mice (Figure 4). However, SDS-PAGE results showed that although there was a significantly greater amount of β-MHC protein in the left ventricle of the D73N (+/−) mice, the amount of β-MHC protein relative to the amount of total MHC was still very low (~3%). The small shift in the relative expression of MHC isoforms is unlikely to affect the Ca^2+^ sensitivity of force development (Fitzsimons et al., 1998; Schoffstall et al., 2006; Suzuki et al., 2009).

Most mutations associated with DCM are inherited in an autosomal dominant pattern (for review Martins et al., 2002; Towbin, 2014). Affected individuals are usually heterozygous for any given mutation, and are expected to express the mutant protein at ~50% or less. Even a modest amount of mutated protein can result in severe pathophysiological consequences. For instance, heterozygous knock-in mice expressing cTnI^R21C^ [linked to hypertrophic cardiomyopathy (HCM)] at only ~25% of total cTnI developed hallmark traits associated with HCM (Wang et al., 2012). None of the homozygous D73N (+/+) mice survived longer than one day. The phenotype of the D73N (+/−) mice resembled that of homozygous knock-in mice harboring the DCM-linked ΔK210 mutation in cTnT (homozygous ΔK210cTnT mice) (Du et al., 2007; Sugihara et al., 2013). Like the D73N (+/−) mice, homozygous ΔK210cTnT mice developed early onset DCM, with decreased Ca^2+^ sensitivity of force development, enlarged hearts, left ventricular dilation, and systolic dysfunction. In addition, electrophysiological abnormalities, such as prolongation of QRS and QT intervals, were observed in both the D73N (+/−) (Figure 7) and homozygous ΔK210cTnT mice. Prolonged QRS intervals likely reflect an increase in left ventricular dimensions and impairment of systolic function (Dhingra et al., 2005) in the D73N (+/−) mice, while prolonged QT intervals are associated with increased risk of sudden cardiac death (for review (Schwartz, 2013; Tester and Ackerman, 2014). However, the phenotype of the D73N (+/−) mice was more severe than that of heterozygous ΔK210cTnT mice. Comparison of results obtained in the current study with that from an earlier study (Liu et al., 2012b) indicates that the D73N mutation in cTnC had a larger effect on the Ca^2+^ sensitivity and rate of Ca^2+^ dissociation from reconstituted thin filaments than the ΔK210 modification in cTnT. Thus, a larger extent of Ca^2+^ desensitization could be responsible for a more severe phenotype of the D73N (+/−) mice compared to heterozygous ΔK210cTnT mice.

In addition to Ca^2+^ desensitization, the D73N mutation blunted the effect of cTnI pseudo-phosphorylation on Ca^2+^ sensitivity and the rate of Ca^2+^ dissociation from reconstituted thin filaments (Figure 1). Furthermore, ventricular myocytes isolated from the D73N (+/−) mice did not respond to β-adrenergic stimulation (Figure 8). In fact, in the presence of a β-adrenergic agonist (ISO), myocytes isolated from the D73N (+/−) mice displayed reduced fractional sarcomere shortening and slower return and departure velocities, compared to that in WT mice (Figure 8 and Table 3). Therefore, in the presence of a β-adrenergic agonist, the D73N mutation resulted in impaired contractility of ventricular myocytes.

Recently, it was hypothesized that blunting or abolishing the extent of Ca^2+^ desensitization (induced by cTnI phosphorylation during β-adrenergic stimulation) is sufficient to trigger the development of DCM (for review Messer and Marston, 2014). In fact, blunting or abolishing of Ca^2+^ desensitization induced by cTnI phosphorylation appears to be a common feature among mutations in cTnC linked to DCM in human patients (with an exception of the D145E mutation) studied to date (Biesiadecki et al., 2007; Pinto et al., 2011). In addition, Ca^2+^ desensitizing effect of cTnI phosphorylation by PKA was abolished in skinned muscle fibers isolated from the left ventricle of homozygous ΔK210cTnT mice (Inoue et al., 2013). However, the “blunting” phenomenon is not unique to DCM, since a number of HCM-linked mutations also blunt the extent of Ca^2+^ desensitization induced by cTnI phosphorylation (Deng et al., 2003; Schmidtmann et al., 2005). Our results show that like cTnI pseudo-phosphorylation, the D73N mutation in cTnC decreased the Ca^2+^ sensitivity of reconstituted thin filaments by accelerating the rate of Ca^2+^ dissociation (Figure 1). Possibly, the D73N mutation and cTnI phosphorylation each result in Ca^2+^ desensitization by destabilizing of the “open” state of the regulatory N-domain of cTnC reconstituted into thin filaments, and shifting the equilibrium toward the “closed” state. Thus, blunted extent of Ca^2+^ desensitization induced by cTnI phosphorylation could be a consequence of already decreased Ca^2+^ sensitivity in thin filaments reconstituted with the cTn^D73N^ complex.

The Ca^2+^ desensitization would lead to hypo-contractile response at rest, while blunted response to β-adrenergic stimulation would lead to hypo-contractile response under stress. Thus, Ca^2+^ desensitization and blunted response to β-adrenergic stimulation could both be contributing to the DCM-like phenotype of D73N (+/−) mice. However, it is likely that Ca^2+^ desensitization is primarily responsible for the severity of the DCM phenotype in the D73N (+/−) mice. For instance, recently generated transgenic mice, which harbored DCM-linked mutation E361G in cardiac actin, did not exhibit a DCM-like phenotype under normal mouse husbandry conditions (Song et al., 2010). While the E361G mutation in actin abolished the Ca^2+^ desensitizing effect of cTnI phosphorylation on cardiac myofibrils (Memo et al., 2013; Vikhorev et al., 2014), skinned papillary muscles isolated from the hearts of transgenic mice, harboring the E361G mutation, did not display a decrease in the Ca^2+^ dependence of force development (Song et al., 2010). Thus, mutations that blunt or abolish the extent of Ca^2+^ desensitization induced by cTnI phosphorylation, but don't desensitize cardiac muscle to Ca^2+^, are not likely to result in a phenotype as severe as occurs in the D73N (+/−) mice.

In conclusion, we generated a novel knock-in mouse model, enabling us to examine physiological consequences of altering Ca^2+^ sensitivity of cTnC. Heterozygous knock-in mice displayed hallmark features of early onset DCM, such as premature death, increased left ventricular dimensions with thinned walls, and impaired systolic function. Our results confirm that abnormal response of the regulatory N-domain of cTnC to Ca^2+^ could have a severe detrimental effect on the normal physiological function of the heart.

## Funding

Research reported in this publication was supported by the NHLBI institute of NIH under Award Number R15 HL117034 (to ST), R01 HL085487 (to BM), and R15 HL124458 (to BM). This project was also supported by the Mouse Phenotyping Core at Baylor College of Medicine with funding from the NIH (U54 HG006348). The content is solely the responsibility of the authors, and does not necessarily represent the official views of the NIH.

### Conflict of interest statement

The authors declare that the research was conducted in the absence of any commercial or financial relationships that could be construed as a potential conflict of interest.
